# Mechanisms of Transcranial Magnetic Stimulation Treating on Post-stroke Depression

**DOI:** 10.3389/fnhum.2018.00215

**Published:** 2018-05-30

**Authors:** Xiaoqin Duan, Gang Yao, Zhongliang Liu, Ranji Cui, Wei Yang

**Affiliations:** Jilin Provincial Key Laboratory on Molecular and Chemical Genetics, The Second Hospital of Jilin University, Changchun, China

**Keywords:** noninvasive brain stimulation, transcranial magnetic stimulation, post-stroke depression, mechanism, BDNF

## Abstract

Post-stroke depression (PSD) is a neuropsychiatric affective disorder that can develop after stroke. Patients with PSD show poorer functional and recovery outcomes than patients with stroke who do not suffer from depression. The risk of suicide is also higher in patients with PSD. PSD appears to be associated with complex pathophysiological mechanisms involving both psychological and psychiatric problems that are associated with functional deficits and neurochemical changes secondary to brain damage. Transcranial magnetic stimulation (TMS) is a non-invasive way to investigate cortical excitability via magnetic stimulation of the brain. TMS is currently a valuable tool that can help us understand the pathophysiology of PSD. Although repetitive TMS (rTMS) is an effective treatment for patients with PSD, its mechanism of action remains unknown. Here, we review the known mechanisms underlying rTMS as a tool for better understanding PSD pathophysiology. It should be helpful when considering using rTMS as a therapeutic strategy for PSD.

## Introduction

Stroke is the most common cause of adult disability in developing countries (Kaadan and Larson, 2017) and has both physical and economic repercussions for patients. Post-stroke depression (PSD) is a severe and fearful complication that occurs in nearly one third of patients who suffer stroke and can even occur in patients who have suffered only a minor stroke or transient ischemic attack (TIA; Carnes-Vendrell et al., 2016). PSD can affect functional ability, rehabilitation outcome, and quality of life, and is related to a higher mortality rate of stroke patients (Miranda et al., 2018). Additionally, stroke severity is an important risk factor for PSD, as is the mental history of the patient. Preventing PSD requires participation from family members and society (Shi et al., 2017). It appears to be associated with complex pathophysiological mechanisms involving both psychological and psychiatric problems that are associated with functional deficits and neurochemical changes secondary to brain damage. Although antidepressants are considered the treatment of choice for PSD, the benefits are not perfect. Indeed, whether pharmacological treatment is needed to prevent PSD or improve neurological outcomes after stroke is uncertain (Kim, 2016; Xu et al., 2016). Fortunately, studies suggest that transcranial magnetic stimulation (TMS) is beneficial for patients with PSD (Gu and Chang, 2017; Shen et al., 2017).

TMS is an important technique for noninvasive brain stimulation (NIBS; Edwards et al., 2017). NIBS, using electromagnetic waves and direct electrical current, is a new frontier in treating neuropsychiatric illnesses or psychiatric maladies (Gupta and Adnan, 2018). Several types of NIBS have been developed over the years, including electroconvulsive therapy (ECT), transcranial alternating current stimulation (tACS), magnetic seizure therapy (MST), TMS and transcranial direct current stimulation (tDCS). Among them, ECT is the best at reducing depression and has unparalleled efficacy even in older populations. However, the risk of amnesia is a severely limiting factor. While tACS has several advantages including biphasic and sinusoidal currents, the ability to entrain large neuronal populations, and subtle control over somatic effects, its best practices remain unclear and further study is required (Tavakoli and Yun, 2017). MST is a proposed form of electrotherapy using magnetic brain stimulation. It preserves the efficacy of ECT while reducing the risk of amnesia through the more precise localization offered by magnetic stimulation (Luber et al., 2013). However, its clinical effects still need to be studied. The most commonly used NIBS are TMS and tDCS. tDCS modulates membrane potential via electrical currents (Rektorová and Anderková, 2017). It does not directly induce action potentials in neurons, but instead is believed to influence spontaneous activity of targeted brain networks. TMS can be directed more specifically than tDCS. Additionally, it can exert a causal influence on brain networks and its clinical efficacy has already been established in the treatment of mental disorders (Hendrikse et al., 2017). Among all the types of NIBS, TMS—especially repetitive TMS (rTMS)—is the best at controlling the frequency and the location of stimulation. This advantage, in addition to others, has opened up new possibilities for clinical exploration and treatment of neuropsychiatric conditions. Meta-analysis of the literature shows that rTMS can combat PSD and that it is actively used in therapy (Klein et al., 2015). However, its exact mechanism of action is still unknown. Here, we explore what we know about the mechanisms underlying rTMS treatment of PSD.

## Mechanisms Through Which TMS Improves PSD

In 2008, rTMS on the left dorsolateral prefrontal cortex (DLPFC) was approved for the treatment of major depression in the USA (Saitoh et al., 2012). Since then, rTMS has been widely used in cases of treatment-resistant depression (TRD) that do not respond adequately to adequate courses of at least two antidepressants (Xie et al., 2013; Lucas et al., 2017). Moreover, application of high frequency rTMS over the dorsal anterior cingulate cortex (dACC) and medial prefrontal cortex (mPFC) has been reported to be a useful intervention for apathy resulting from stroke (Sasaki et al., 2017). A few related studies have examined the mechanism through which rTMS lessens depression in PSD. TMS is known to influence neuronal plasticity in the brain by originating the long-term potentiation (LTP) and long-term depression (LTD). Low-intensity TMS primarily stimulates low-threshold inhibitory neurons, while high-intensity TMS excites projection neurons. Although detailed mechanisms have not yet been characterized very well, several theories have been proposed.

### Increased Concentration of Brain-Derived Neurotrophic Factor (BDNF)

Brain-Derived Neurotrophic Factor (BDNF) is an important neurotrophic factor that is distributed extensively in the central nervous system. BDNF is crucial for the survival, growth and maintenance of neurons within brain circuits that control emotion and cognition (Kowiański et al., 2018). Phillips (2017) has suggested that neuroplasticity that takes place in major depressive disorder (MDD) is related to BDNF levels. BDNF is crucial for exercise learning and systemic rehabilitation after stroke. BDNF concentration in patients with acute ischemic stroke has been reported to be lower than that in healthy controls. Additionally, low levels of BDNF are associated with increased risk of stroke, worsening of functional outcome, and increased stroke-related mortality. Recently, single nucleotide polymorphisms of the BDNF gene were studied in patients with stroke. Mizui et al. (2017) found that the BDNF pro-peptide can facilitate hippocampal LTD and that the BDNF polymorphism Val66Met impacted the biological activity of the BDNF pro-peptide. Further, Kotlęga et al. (2017) reported that the Met allele is associated with adverse consequences and prognosis after stroke. Thus, BDNF and BDNF polymorphisms play important roles in brain plasticity, especially in changes of function and morphology.

PSD has been correlated with low BDNF expression levels (Chen et al., 2015). Low levels of BDNF can lead to mood dysregulation and loss of hippocampal function. A variety of biological, environmental and pharmacological factors can affect mood by modulating BDNF expression (Phillips, 2017). Additionally, studies have shown that high-frequency rTMS enhances BDNF expression levels. BDNF is a key factor in the increased hippocampal cell proliferation and neuronal differentiation after application of rTMS. Further, reports show that in several brain areas in rats, including the hippocampal CA1 and CA3 subfields, high-frequency rTMS (20 Hz) triggers BDNF expression. Aside from increasing BDNF levels, rTMS might also activate the BDNF/ERK signaling pathway to upregulate cell proliferation in the hippocampus (Cui et al., 2017).

### Increased Glucose Metabolism in the Cortex and in Specific Neural Networks

In a seminal experiment, Siebner et al. (1998) measured the relative changes in the regional cerebral metabolic rate of glucose (rCMRglc) during 2 Hz rTMS of the left sensorimotor cortex and during imitation of rTMS-induced arm movements using positron emission tomography (PET). Since then, combining rTMS and PET has been used to visualize rTMS-related net brain activation and to analyze functional networks (Li et al., 2013; Val-Laillet et al., 2015; Heiss, 2016).

More recently, Parthoens et al. (2014) combined the neurostimulation with positron emission tomography (microPET) in small animals to quantify regional 2-Deoxy-2-[^18^F] fluoro-D-glucose ^18^F-FDG uptake in the rat brain in response to low- (1 Hz) or high- (50 Hz) frequency paradigms. This method is now often used to visualize neuronal activation of the cortex and cortical networks during different types of stimulation (Akman et al., 2015; Adriaanse et al., 2016; Malpetti et al., 2017). rTMS at differing frequencies has been shown to decrease ^18^F-FDG-uptake in dorsal cortical regions while increasing it in ventral regions (Parthoens et al., 2016). Baeken et al. (2015) examined the clinical effects of high-frequency rTMS on glucose metabolism in the subgenual anterior cingulate cortex (sgACC) by stimulating left DLPFC. The results show that sgACC is a specific region whose treatment with high frequency rTMS leads to an antidepressant response. Another study demonstrated that the clinical response to high frequency-rTMS treatment in patients with TRD might depend on the metabolic state of the cerebellum. It implied that other localized brain regions might be warranted for stimulation, especially the cerebellum when patients do not respond to DLPFC high-frequency rTMS (Wu and Baeken, 2017). In a related ^18^F-FDG-PET study, an increase in glucose metabolism of the rat cerebellar cortex was observed after high-frequency rTMS, along with reduced synthesis of neural plasticity-related proteins such as metabotrophic glutamate receptor (mGluR), protein kinase C (PKC) and glutamate receptor 2 (GluR2). These findings suggested that rTMS could act on the rat cerebellar cortex to induce LTD-related neuroplasticity (Lee et al., 2014). Another study suggests that manipulating pre-rTMS neural activity in patients with MDD could predict and augment the antidepressant effects of rTMS treatment, including increased frontal θ and glucose uptake (Li et al., 2016). Some scholars have used rTMS with ^18^F-FDG-PET to study the neurophysiological and spatial dynamics induced by repetitive 1-Hz rTMS in the temporal cortex. One study found that rTMS can cause a reduction in glucose metabolism of the temporal lobe and an increase in glucose metabolism of the mPFC and ipsilateral cortex. Further, statistical parametric mapping of FDG-PET data revealed a focal reduction of glucose metabolism in the stimulated temporal area and an increase in the bilateral precentral, ipsilateral superior and middle frontal, prefrontal, and cingulate gyri. This suggests that 1-Hz rTMS in the temporal cortex can cause cortico-cortical modulation and induce extensive functional changes in neural networks via long-range neuronal connections (Lee et al., 2013). Hayashi et al. (2004) showed statistically robust changes in FDG uptake in the macaque brain after the rTMS. Specifically, they found a reduction in the motor/premotor cortices and an increase in orbitofrontal cortices and the limbic-associated areas involving the anterior/posterior cingulate. Impressively, these changes in uptake continued for at least 8 days after treatment. These results demonstrate that functional connections allow motor rTMS to have long-lasting effects on motor-related regions and distant limbic-related areas.

Glucose metabolism is known to decrease in the ischemic hemisphere, especially in patients with PSD. Using ^18^F-FDG micro-PET imaging, Gao et al. (2010) reported that glucose uptake in rat cortex and striatum was larger in an rTMS group than in a control group. Meanwhile, the number of caspase3-positive cells was significantly lower, and the ratio of Bcl-2/Bax was higher in the rTMS group. Thus, rTMS treatment increased glucose metabolism and inhibited apoptosis in the ischemic brain. Additionally, rTMS has emerged as a promising therapeutic intervention in the treatment of affective disorders. Preliminary evidence from PET scans suggests that high-frequency (20 Hz) stimulation might increase brain glucose metabolism in a transsynaptic fashion, whereas low-frequency (1 Hz) stimulation might do the opposite (Post et al., 1999). Another study has shown that glucose levels decreased after tDCS (Sampaio et al., 2012). No study has reported a change in glucose levels after non-repeated TMS. In the future, our team intends to explore glucose levels in patients with PSD who have diabetes.

### Increased Neurogenesis

Neuroplasticity is a natural property of the nervous system that allows functional changes and reorganization after lesions or environmental changes. PSD has been shown to be linked to basal ganglia and frontal lobe lesions, white matter degeneration, and interruption of brain network connectivity. TMS has been reported to increase white matter fractional anisotropy (FA) values and increase left frontal lobe activation (Hallett et al., 2017). Also, TMS can induce electro-magnetic currents in the related cortical neurons. Varying frequencies and intensities of TMS can directly increase or decrease excitability in the cortical area (Ambriz-Tututi et al., 2012) so as to promote the functional reconstruction of the damaged neural network and repair neuronal structure. Thus TMS can contribute to brain-network research including the study of cortical-cerebellar and cortical-basal ganglia relationships. Furthermore, it was reported that rTMS is conductive to the proliferation, differentiation and migration of neural stem cells (NSCs) and inhibits their apoptosis (Cui et al., 2017). Different magnitudes and numbers of pulses can induce different effects on NSCs, meanwhile, several neurotransmitter systems, such as the γ-aminobutyric acid (GABA) system, could be activated by rTMS to modulate the niche of NSCs in the subventricular zone (or other region with NSCs) to cause an increase in cell proliferation (Lozeron et al., 2016; Cui et al., 2017). Indeed, when rats with post-traumatic brain injury received rTMS, they exhibited significantly greater proliferation in the subventricular zone, significantly higher rates of neuron survival, and significantly reduced rates of apoptosis than similarly injured control rats (Lu et al., 2017). These findings suggest that high-frequency rTMS could promote neurogenesis.

### Modulation of Neurobiochemical Effects

Stroke can lead to abnormalities in the expression of biogenic amine neurotransmitters and cytokines. Additionally, reactive oxygen species generated during stroke can cause oxidative stress, lipid peroxidation and protein oxidation in nerve tissue. PSD could be related to any of these pathophysiological processes (Nabavi et al., 2015).

Experimental evidence in rodents indicate that rTMS produces complex neurobiochemical effects such as induction of immediate early genes, changes in how neurotransmitters release is modulated, changes in glutamate α-amino-3-hydroxy-5-methyl-4-isoxazolepropionic acid (AMPA) receptor/N-methyl-D-aspartate (NMDA) receptor expression (influencing calcium ion dynamics), actions on the neuroendocrine system, neuroprotection via reduced oxidative stress and inflammation, and changes in neurotrophin expression. These molecular effects may modify the intrinsic and extrinsic electrophysiological properties of neurons and reprogram the expression of excitatory and inhibitory neurotransmitters and their cognate receptors, which lead to long-lasting synaptic plasticity-related changes similar to LTP and LTD (Di Lazzaro et al., 2010; Soundara Rajan et al., 2017).

EI Arfani et al. (2017) found that total striatal 5-hydroxyindolacetic acid (a metabolite of serotonin) levels were reduced after accelerated high-frequency rTMS, and motor activity in rats increased as a result. In addition to its clinical antidepressant effect, serotonin can improve motor function and assist learning and memory functions. Studies have shown that serotonin can induce neural plasticity by modulating paired co-stimuli, which may help explain the mechanism by which serotonin plays a positive role in learning and as a medical treatment for depression and stroke (Batsikadze et al., 2013).

In addition, applying rTMS to the motor cortex increases dopamine in the striatum (Kulishova and Shinkorenko, 2014), and improved motor performance in PD may be related to an elevation of serum dopamine concentration after rTMS (Khedr et al., 2007). Strafella et al. (2003) also measured changes in extracellular dopamine concentration following rTMS of the motor cortex and found that it led to focal dopamine release in the ipsilateral caudate nucleus. Further, rTMS is capable of inducing lasting alterations in cortical excitability (Liebetanz et al., 2003). Thus, rTMS can increase cortical excitability via dopamine release. It is worthy to be studied in future.

### Regulation of Emotion Through LTD-Like and LTP-Like Plasticity

rTMS can regulate emotion through both inhibition and excitation. A newer form of rTMS protocol, known as theta-burst stimulation (TBS), has been shown to produce similar if not greater effects on brain activity than standard rTMS (Chung et al., 2015). Inhibitory rTMS is thought to act via LTD and require low-frequency (1 Hz) stimulation and continuous theta burst stimulation (cTBS). In contrast, excitatory rTMS is thought to act via LTP, require high-frequency (5–20 Hz) stimulation and intermittent theta burst stimulation (iTBS). One study suggests that rTMS may be able to effectively and selectively modulate psychiatric symptomatology in which the orbitofrontal cortex (OFC) is implicated (Fettes et al., 2017). Depressed patients have hypometabolism of the left DLPFC, which is ameliorated by rTMS. rTMS regulates mood by acting on the cortical-subcortical network (Shen et al., 2017). Casula et al. (2016) found that the DLPFC has the potential to generate robust spike-timing dependent plasticity (STDP). Pellicciari et al. (2017) reported that bilateral TBS treatment induced a remarkable rearrangement of bilateral DLPFC oscillatory activity, which parallels clinical advances. Specifically, left DLPFC iTBS decreased θ- and α-band oscillations and increased higher frequencies, while right DLPFC cTBS increased α-oscillatory activity evoked by TMS. These results highlighted that idea that iTBS is able to modulate cortical excitability and increase spontaneous neuronal activity in the higher frequency ranges. Additionally, priming TBS of rTMS is ineffective in modifying motor cortex (M1) plasticity in older adults because the neuroplastic potential of primary motor cortex in older adults is low (Opie et al., 2017). Research on younger individuals suggests that neuroplastic responses can be enhanced via rTMS, with larger responses observed following both LTP and LTD-like protocols. In this sense, the effect of rTMS on neuroplasticity is related to age. Thus, rTMS can improve cortical excitation in patients with PSD, which can improve their emotion.

## Conclusions and Future Directions

Although the mechanisms underlying the effect of rTMS on PSD are unknown, its effectiveness cannot be denied. In our clinical work, we generally use 1000 rTMS pulses (5–10 Hz at 80%–100% of resting motor threshold [rMT]) over the left DLPFC and 1000 rTMS pulses (1 Hz at 80%–100% of rMT) over the right DLPFC for 10 days to treat depression after stroke. Understanding the how rTMS affects PSD should help the development of new and more effective treatments, and the mechanism should be further studied to provide a strong theoretical basis for clinical application.

Recently, researchers have explored the mechanisms underlying rTMS therapy using a variety of methods. Neuroimaging, including PET and functional magnetic resonance imaging, have been used to learn about the remote effects of TMS on the brain (Hallett et al., 2017). Also, TMS-induced electromyographic (EMG) and electroencephalographic responses to drugs can help us understand excitability, connectivity and plasticity in brain (Ziemann et al., 2015). Based on the thinking that motor-evoked potentials elicited by TMS in a target muscle are variable, and that the source of variable muscle responses may not be apparent using conventional bipolar EMG (particularly over areas with several distinct neighboring muscles), Neva et al. (2017) suggest that high-density surface EMG (HDsEMG) provides a useful way to differentiate which wrist extensor muscles are activated by TMS. In future studies, a record of event-related potentials combined with machine learning and applied statistics could be used to build models of neural activity (Holdgraf et al., 2017) that will reveal more details regarding the underlying mechanisms of rTMS-related improvement in PSD.

## Author Contributions

XD and GY wrote the manuscript. ZL, WY and RC provided the critical revisions. All authors approved the final version of the manuscript for submission.

## Conflict of Interest Statement

The authors declare that the research was conducted in the absence of any commercial or financial relationships that could be construed as a potential conflict of interest.
